# Sortilin and its potential role in cardiovascular pathology

**DOI:** 10.1186/s43044-024-00512-3

**Published:** 2024-06-24

**Authors:** Alim Namitokov

**Affiliations:** 1 Kuban State Medical University, Krasnodar, Russia; 2 Scientific Research Institute – Regional Clinical Hospital #1 NA Prof. S.V. Ochapovsky, Krasnodar, Russia

## Abstract

**Background:**

This comprehensive review explores the multifaceted role of sortilin, a key receptor in lipid metabolism, within the context of cardiovascular diseases (CVDs), the leading cause of global mortality.

**Main body:**

Sortilin, encoded by the SORT1 gene, is implicated in the pathogenesis of atherosclerosis, primarily through its regulation of low-density lipoprotein cholesterol (LDL-C) and very low-density lipoproteins (VLDL). The review delves into the biological functions of sortilin, emphasizing its critical role in lipid and cholesterol homeostasis and its influence on hepatic secretion of lipoproteins and atherogenesis. We highlight sortilin's pathophysiological significance in atherosclerosis, underscoring its involvement in lipid metabolism pathways and vascular inflammation, and its impact on macrophage functions in atherosclerotic plaque formation. The potential of sortilin as a therapeutic target is discussed, considering evidence that suggests its modulation could ameliorate atherosclerosis. The review also acknowledges current inconsistencies and gaps in the evidence, calling for more comprehensive patient studies and in-depth mechanistic research. Finally, the article outlines future research directions, focusing on understanding sortilin's specific cellular mechanisms in cardiovascular health, exploring its genetic variability, therapeutic implications, and its broader relevance to other diseases.

**Conclusion:**

This review underscores the significance of sortilin as a biomarker and a promising target for therapeutic intervention in cardiovascular pathology, while advocating for continued research to fully unravel its complex role.

## Background

Cardiovascular diseases (CVDs) are the number one cause of death globally, representing a major issue in both high-income and developing countries [1]. These include a variety of disorders affecting the heart and blood vessels, such as coronary heart disease, cerebrovascular disease, and hypertension. The global impact is significant, with millions of people dying annually from CVDs, often due to a combination of behavioral risk factors (like tobacco use, unhealthy diet, and physical inactivity) that lead to raised blood pressure, glucose, and lipid levels [2–4]. Given this backdrop, understanding the biological mechanisms and potential biomarkers like sortilin is crucial for developing targeted interventions to reduce the burden of CVDs [5]. Sortilin is a protein that has garnered attention in cardiovascular research due to its multifunctional roles. It is involved in regulating cholesterol metabolism, particularly by affecting the levels of low-density lipoprotein (LDL) cholesterol, known to contribute to atherosclerosis. Sortilin also influences the secretion of very low-density lipoproteins (VLDL) and the activity of PCSK9, a protein that degrades LDL receptors. Beyond lipid metabolism, sortilin is implicated in the pathogenesis of vascular inflammation and plaque formation. The relevance of sortilin in cardiovascular health is underscored by the genetic association of SORT1 gene variations with an increased risk of coronary artery disease. Understanding sortilin’s pathways offers potential for new therapeutic strategies to combat cardiovascular diseases [5–8].

## Main text

### The biology of sortilin

Sortilin is a multifunctional receptor involved in various cellular processes, including intracellular sorting, regulation of enzyme and neuropeptide transport, and signaling. It is a type I membrane protein encoded by the SORT1 gene. Structurally, sortilin has a large luminal domain, a single transmembrane region, and a short cytoplasmic tail. Functionally, it binds to various ligands, directing them to different cellular pathways. In the context of cardiovascular health, sortilin regulates lipoprotein metabolism and is implicated in the pathogenesis of atherosclerosis due to its role in lipid and cholesterol homeostasis [8, 9].

The SORT1 gene, located on chromosome 1p13.3, encodes the protein sortilin. Genetic studies have identified this locus as a significant predictor of LDL cholesterol levels and, consequently, a risk factor for coronary artery disease. Variants in the SORT1 gene have been associated with changes in the expression of sortilin, influencing lipid metabolism and atherosclerosis development [10].

### Sortilin in lipid metabolism and it’s potential role in atherosclerosis

The SORT1 gene has been shown to influence the metabolism of lipoproteins, particularly LDL-C and VLDL. It affects the plasma levels of these lipids, which are critical in the development of atherosclerosis and cardiovascular diseases [10].

Sortilin has been identified as a key player in the regulation of lipid metabolism, specifically in the hepatic secretion of lipoproteins. It interacts with apolipoprotein B100 (apoB100), a primary component of LDL and VLDL particles. Through this interaction, sortilin mediates the hepatic uptake and degradation of lipoproteins, thus influencing plasma LDL-C levels, which are a well-established risk factor for cardiovascular diseases. Additionally, sortilin impacts the secretion of VLDL, which is an important precursor to LDL, further establishing its role in lipid homeostasis and atherogenesis [8].

Sortilin's role in lipid metabolism is facilitated through its interaction with apoB100, which is integral to the formation and secretion of LDL particles. Sortilin influences the hepatic release of apoB100-containing lipoproteins, thus affecting circulating LDL levels [11].

Sortilin's pathophysiological role in atherosclerosis is multifaceted, involving distinct pathways in lipoprotein metabolism and vessel wall inflammation [12]. It has been linked to atherosclerosis through its association with hyperlipidemia and is believed to affect the function of peripheral macrophages, which are critical in the development of atherosclerotic plaques [13].

Targeting sortilin in immune cells can reduce proinflammatory cytokines and atherosclerosis. This has been supported by genome-wide association studies identifying a link between genetic variation at the human chromosomal locus 1p13.3, where the SORT1 gene is located, and coronary artery disease [14]. Moreover, sortilin contributes to vascular calcification in atherosclerosis by externalizing alkaline phosphatase-containing vesicles, linking it to cellular senescence [15].

Research has shown that the SORT1 gene within the 1p13.3 locus is an important modulator of LDL-cholesterol level and atherosclerosis risk. The effects of SORT1 on lipid metabolism and development of atherosclerosis have been explored, particularly focusing on sortilin's effects in hepatocytes and macrophages. Hepatic sortilin overexpression can lead to both increased and decreased LDL-C levels by several different mechanisms, indicating complex roles of sortilin in hepatic lipid metabolism. In macrophages, sortilin causes internalization of LDL and probably a reduction in cholesterol efflux, leading to intracellular accumulation of excessive lipids. A sortilin deficiency in an atherosclerotic mouse model results in decreased aortic atherosclerotic lesions, suggesting that sortilin promotes the development of atherosclerosis and could be a potential therapeutic target for treatment [16].

Another study demonstrates that macrophage sortilin deficiency protects against atherosclerosis by reducing macrophage uptake of LDL. Sortilin-mediated uptake of native LDL into macrophages may be an important mechanism of foam cell formation and contributor to atherosclerosis development. In experiments, sortilin-deficient macrophages showed significantly reduced uptake of native LDL ex vivo and reduced foam cell formation in vivo. In contrast, overexpression of sortilin in macrophages resulted in increased LDL uptake and foam cell formation [17].

**Fig. 1:**
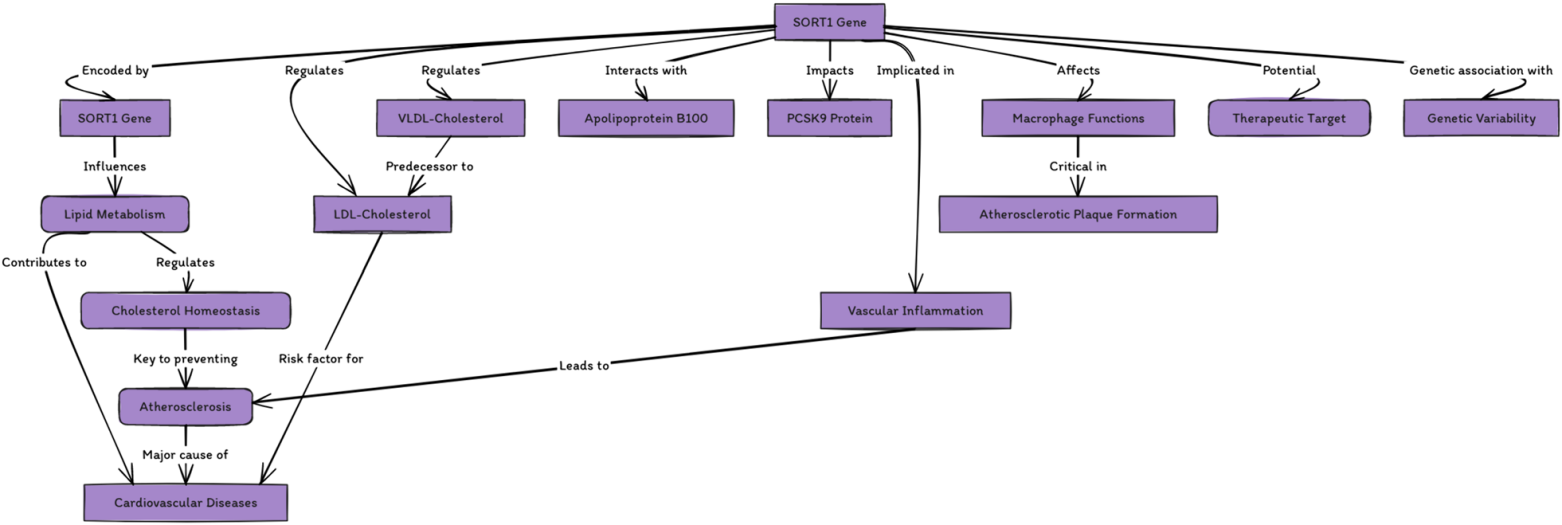
Central illustration

This evidence collectively underscores sortilin's significant role in atherosclerosis and points to its potential as a therapeutic target in atherosclerosis treatment (Fig. 1).

Plasma LDL-C levels are a significant risk factor for cardiovascular disease, and the SORT1 gene at the 1p13 locus has been associated with changes in LDL-C levels and cardiovascular risk. This study highlights the work of three independent teams that identified SORT1 as a gene involved in LDL metabolism. Despite using different methods, these teams provided essential insights into the role of sortilin in cholesterol regulation, albeit with some contradictory results. This research has opened new avenues for understanding and potentially targeting cardiovascular disease [7].

A study aimed to evaluate sortilin as a biomarker for coronary artery disease (CAD) within a well-characterized cohort. This study enrolled 1,173 patients with suspected stable CAD and measured sortilin using two different methods: ELISA and OLINK Cardiovascular Panel II. The results showed a poor correlation between the methods. Furthermore, the study found no association between plasma sortilin levels and traditional CAD risk factors or disease severity. It concluded that sortilin might not be a useful biomarker for CAD in a clinical setting for patients with low to intermediate risk [6].

In a study exploring the relationship between sortilin and inflammation in coronary heart disease (CHD), elevated levels of sortilin were observed in CHD patients, and these levels were significantly positively correlated with proinflammatory cytokines. The study also identified sortilin polymorphisms associated with the occurrence of CHD. This suggests that sortilin may interact with inflammatory responses to contribute to the development of CHD, indicating that its role in CHD's inflammatory processes warrants further investigation [18].

This finding, along with previous research that identifies sortilin's involvement in lipid metabolism and atherogenesis, provides a multifaceted view of sortilin's role in cardiovascular disease. However, the current evidence also shows inconsistencies and gaps that need to be addressed through more extensive and diverse patient studies, as well as mechanistic explorations into sortilin's role in cardiovascular pathology.

### Future perspectives and research directions

Certainly, identifying unanswered questions and suggesting future research areas is essential for advancing our understanding of sortilin's role in cardiovascular diseases. Here are some points that could be addressed:

- Mechanistic Action: What specific cellular mechanisms does sortilin engage in within the cardiovascular system? How does it interact with other proteins and receptors involved in cardiovascular health and disease?
- Genetic Variability: How do different genetic variants of the SORT1 gene influence the function of sortilin and the risk of developing cardiovascular diseases across different populations?
- Therapeutic Targeting: Can sortilin be effectively targeted by pharmaceuticals to reduce the risk or severity of cardiovascular diseases? What would be the potential side effects of such treatments?
- Prognostic Potential: Could sortilin levels be used as a prognostic tool for cardiovascular diseases? How can this be standardized across clinical settings?
- Interaction with Other Biomarkers: How does sortilin interact with other established biomarkers of cardiovascular disease? Can a multi-marker approach improve risk assessment and patient outcomes?
- These areas are ripe for exploration and could significantly contribute to the field of cardiovascular research.

Regarding the development of drugs aimed at correcting sortilin, one notable study reports the development of TH1902, a docetaxel-peptide conjugate for the treatment of sortilin-positive triple-negative breast cancer. This conjugate utilizes sortilin's function in ligand internalization to selectively target and kill cancer cells expressing sortilin. The study demonstrated potent antiproliferative and apoptotic activities of TH1902 in vitro and greater tumor regression in vivo compared to docetaxel alone, suggesting the potential of sortilin as a target for personalized therapy in cancers where sortilin is overexpressed [19]. Key studies examining sortilin are listed in Table 1.

**Table 1:** Descriptive table of studies

Study	Key findings	Clinical implications
Musunuru et al. [10]	Identified SORT1 as a gene involved in LDL metabolism; highlighted genetic variants affecting LDL cholesterol levels	Insights into genetic risk factors for coronary artery disease; potential for personalized treatment strategies
Mortensen et al. [12]	Link between genetic variation at 1p13.3 locus (SORT1 gene location) and coronary artery disease; Sortilin's role in lipid metabolism and atherosclerosis	Indicates the complex role of sortilin in cardiovascular health; underscores the importance of targeted therapeutic strategies
Patel et al. [17]	Sortilin promotes LDL uptake and foam cell formation in macrophages; implications for atherosclerosis development	Provides a mechanism for the development of atherosclerotic plaques; potential target for atherosclerosis treatment
Møller et al. [6]	Sortilin may not be a useful biomarker for CAD in patients with low to intermediate risk; poor correlation between measurement methods	Highlights the need for further research to establish sortilin's utility as a biomarker in cardiovascular disease
Demeule et al. [19]	Development of TH1902, a sortilin-targeted therapy for triple-negative breast cancer; potential implications for cardiovascular disease therapeutics	Demonstrates the potential of targeting sortilin in therapeutic applications; relevance for both oncology and cardiology

This finding could have implications for cardiovascular diseases if similar principles are applied to develop therapeutics that target sortilin pathways involved in lipid metabolism and atherosclerosis. However, the translation of these findings from oncology to cardiology would require extensive research and clinical trials to understand the safety, efficacy, and potential side effects of such therapies in cardiovascular patients (Table 2).

**Table 2:** Summary table highlighting the key aspects of Sortilin's role in cardiovascular pathology

Aspect	Details
Background	Sortilin, a key receptor in lipid metabolism, plays a role in cardiovascular diseases, the leading cause of global mortality
Main function	Sortilin, encoded by the SORT1 gene, is involved in intracellular sorting, regulation of enzyme/neuropeptide transport, and signaling
Genetic association	Variants in the SORT1 gene are associated with changes in LDL cholesterol levels, affecting the risk of coronary artery disease
Role in atherosclerosis	Sortilin is implicated in atherosclerosis, primarily through its regulation of LDL cholesterol and VLDL
Influence on lipoprotein metabolism	Sortilin regulates the hepatic secretion of lipoproteins, interacting with apolipoprotein B100 and affecting plasma LDL-C levels
Impact on cardiovascular diseases	Sortilin’s modulation could ameliorate atherosclerosis, making it a significant biomarker and potential target for cardiovascular pathology
Therapeutic potential	Evidence suggests sortilin's modulation could lead to new therapeutic strategies for cardiovascular diseases
Current research gaps	Current research presents inconsistencies and gaps in understanding sortilin’s biomarker role, necessitating more extensive studies
Future research directions	Future research should focus on sortilin's specific cellular mechanisms, genetic variability, and therapeutic implications in cardiovascular health

### Key home messages

1. Sortilin's Central Role in Lipid Metabolism: Sortilin, through the SORT1 gene, is intricately involved in the metabolism of LDL cholesterol (LDL-C) and VLDL, substances central to the development of atherosclerosis, a leading cause of cardiovascular disease.
2. Sortilin as a Therapeutic Target: the protein's regulation of lipid levels and impact on inflammatory processes in atherosclerosis presents it as a promising target for therapeutic intervention, potentially offering a novel approach to combat cardiovascular diseases.
3. Need for Comprehensive Research: current studies present inconsistencies regarding Sortilin's role as a biomarker in cardiovascular diseases, highlighting the need for larger, more diverse patient studies and detailed mechanistic research.
4. Future Potential in Treatment and Diagnosis: it anticipates future research that could clarify Sortilin's function, improve cardiovascular risk assessment, and potentially lead to targeted treatments, leveraging its biomarker capabilities.

## Conclusion

Through its involvement in lipid metabolism and inflammatory processes, sortilin emerges as a significant biomarker and potential therapeutic target in cardiovascular pathology. The evidence presented highlights the importance of understanding sortilin's multifaceted functions, from lipid transport to inflammatory responses in the vascular system. However, the review also brings attention to the existing gaps in knowledge and inconsistencies in the current research, emphasizing the need for more comprehensive and detailed studies. These studies should focus on the mechanistic aspects of sortilin's action, its genetic variability, and the implications of targeting sortilin in clinical settings. Furthermore, the potential cross-application of findings from sortilin-targeted therapies in other diseases like cancer to cardiovascular diseases presents an intriguing avenue for future research. In summary, the review not only consolidates the current understanding of sortilin's role in cardiovascular diseases but also sets the stage for future investigations that could lead to more effective and targeted treatments for these globally prevalent conditions.

## Data Availability

Not applicable.

## Abbreviations

ApoB100: Apolipoprotein B100
CAD: Coronary artery disease
CVD: Cardiovascular disease
LDL-C: Low-density lipoprotein cholesterol
VLDL: Very low-density lipoproteins
